# High-dose fasudil preserves postconditioning against myocardial infarction under hyperglycemia in rats: role of mitochondrial KATP channels

**DOI:** 10.1186/1475-2840-11-28

**Published:** 2012-03-22

**Authors:** Taiga Ichinomiya, Sungsam Cho, Ushio Higashijima, Shuhei Matsumoto, Takuji Maekawa, Koji Sumikawa

**Affiliations:** 1 Department of Anesthesiology, Nagasaki University School of Medicine, Nagasaki, Japan; 2 Department of Anesthesiology, Nagasaki University School of Medicine, 1-7-1 Sakamoto, Nagasaki 852-8501, Japan

**Keywords:** Hyperglycemia, Postconditioning, Myocardial Infarction, Fasudil, Mitochondrial KATP channels

## Abstract

**Background:**

The current study was carried out to determine whether fasudil hydrochloride (fasudil), a Rho-kinase inhibitor, has myocardial postconditioning (PostC) activity under hyperglycemia as well as normoglycemia, and if so, whether the effects could be mediated by mitochondrial ATP-sensitive potassium (m-KATP) channels.

**Methods:**

Male Sprague-Dawley rats were anesthetized with sodium pentobarbital. After opening the chest, all rats underwent 30-min coronary artery occlusion followed by 2-h reperfusion. The rats received low-dose (0.15 mg/kg) or high-dose (0.5 mg/kg) fasudil or diazoxide, an m-KATP channel opener, at 10 mg/kg, just before reperfusion under normoglycemic or hyperglycemic conditions. In another group, rats received 5-hydroxydecanoic acid (5HD), an m-KATP channel blocker, at 10 mg/kg, before high-dose fasudil. Myocardial infarct size was expressed as a percentage of area at risk (AAR).

**Results:**

Under normoglycemia, low-dose and high-dose fasudil and diazoxide reduced myocardial infarct size (23 ± 8%, 21 ± 9% and 21 ± 10% of AAR, respectively) compared with that in the control (42 ± 7%). Under hyperglycemia, low-dose fasudil (40 ± 11%) and diazoxide (44 ± 14%) could not exert this beneficial effect, but high-dose fasudil reduced myocardial infarct size in the same manner as under normoglycemia (21 ± 13%). 5HD prevented fasudil-induced reduction of myocardial infarct size (42 ± 13%).

**Conclusion:**

Fasudil induces PostC against myocardial infarction via activation of m-KATP channels in the rat. Although hyperglycemia attenuates the PostC, high-dose fasudil can restore cardioprotection.

## Introduction

Rho-kinase, a ubiquitously expressed serine-threonine protein kinase that is involved in diverse cellular functions, may play a pivotal role in cardiovascular diseases such as vasospastic angina, ischemic stroke and heart failure [1]. Some reports showed that fasudil hydrochloride (fasudil), a Rho-kinase inhibitor, had beneficial effects in ischemic heart disease. A double-blind placebo-controlled trial showed that fasudil increased the ischemic threshold of angina patients during exercise with a trend toward increased exercise duration [2]. Intracoronary administration of fasudil increased oxygen saturation in coronary sinus vein, and ameliorated pacing-induced myocardial ischemia in patients with effort angina [3]. Rho-kinase was activated in ischemic myocardium, and a Rho-kinase inhibitor lead to the activation of the phosphatidylinositol 3-kinase (PI3K)/Akt/eNOS signaling pathway, resulting in preconditioning (PreC) or postconditioning (PostC) [4,5]. Furthermore, hydroxyfasudil was also reported to activate ATP-sensitive potassium (KATP) channels via inhibition of Rho-kinase during myocardial remote peri-conditioning [6]. However, it is not clear whether mitochondrial KATP (m-KATP) channels mediate the Rho-kinase inhibitor-induced PostC.

Hyperglycemia is an important predictor and a risk factor of cardiovascular morbidity and mortality in patients with and without diabetes mellitus [7-9]. A number of studies have shown that the cardioprotective effects of pharmacological PreC and PostC are impaired by hyperglycemia [10-16]. Although the detailed mechanisms are unclear, it has been suggested that the loss of cardioprotection by hyperglycemia might be associated with several mechanisms, namely, generation of large quantities of reactive oxygen species (ROS), inhibition of protective signaling mechanisms, down-regulation of endothelial nitric synthase (eNOS), reduced activation and availability of protective molecules, coronary vascular endothelial dysfunction, and/or impairment in the function of m-KATP channels [11-13,17,18]. However, the effect of hyperglycemia on the Rho-kinase inhibitor-induced myocardial protection against ischemic reperfusion injury is unknown.

The present study was carried out to clarify whether fasudil has PostC activity and, if so, whether the cardioprotective effect is mediated by m-KATP channels and attenuated by hyperglycemia.

## Materials and methods

All experimental procedures and protocols described in this study were approved by the Institutional Animal Care and Use Committee of Nagasaki University School of Medicine.

### Drugs

Fasudil was purchased from Asahikasei Pharma (Tokyo, Japan). 5-hydroxydecanoic acid (5-HD), an m-KATP channel blocker, diazoxide, an m-KATP channel opener, patent blue dye, and 2,3,5-triphenyltetrazolium chloride (TTC) were purchased from Sigma (St. Louis, MO, USA).

### General preparation

The instrumental methods used were as described in our previous report [19]. Male Sprague-Dawley (SD) rats weighing between 350 and 550 g were anesthetized with sodium pentobarbital (a 50 mg/kg intraperitoneal bolus followed by an intravenous infusion of 10-20 mg/kg/h). The rats were adequately sedated to ensure that pedal and palpebral reflexes were absent throughout the experimental protocol. Catheters were inserted into the right jugular vein for fluid or drug administration and the right carotid artery for measurement of arterial blood pressure. After tracheotomy had been performed, the trachea was intubated with a cannula connected to a small animal ventilator (SAR-830 CWE, PA, USA), and the lungs were ventilated with pure oxygen. Arterial blood gas pH was maintained within a physiological range by adjusting the respiratory rate and tidal volume throughout the experiment. A left thoracotomy was performed in the fifth intercostal space, and the pericardium was opened. A 7-0 prolene ligature was placed around the proximal left anterior descending coronary artery (LAD) and vein in the area immediately below the left atrial appendage. The ends of the suture were threaded through a small plastic tube to form a snare for reversible LAD occlusion. Coronary artery occlusion was produced by clamping the snare onto the epicardial surface of the heart and was confirmed by the appearance of epicardial cyanosis. Reperfusion was achieved by loosening the snare and was verified by observing an epicardial hyperemic response. Hemodynamics were continuously monitored with a transducer (sck-9082, Becton Dickinson, Tokyo, Japan) and a blood pressure amplifier (AP-641 G, Nihon-Kohden, Tokyo, Japan) and shown on a polygraph system (RMP-6018, Nihon-Kohden, Tokyo, Japan).

### Experimental protocol

**Figure 1:**
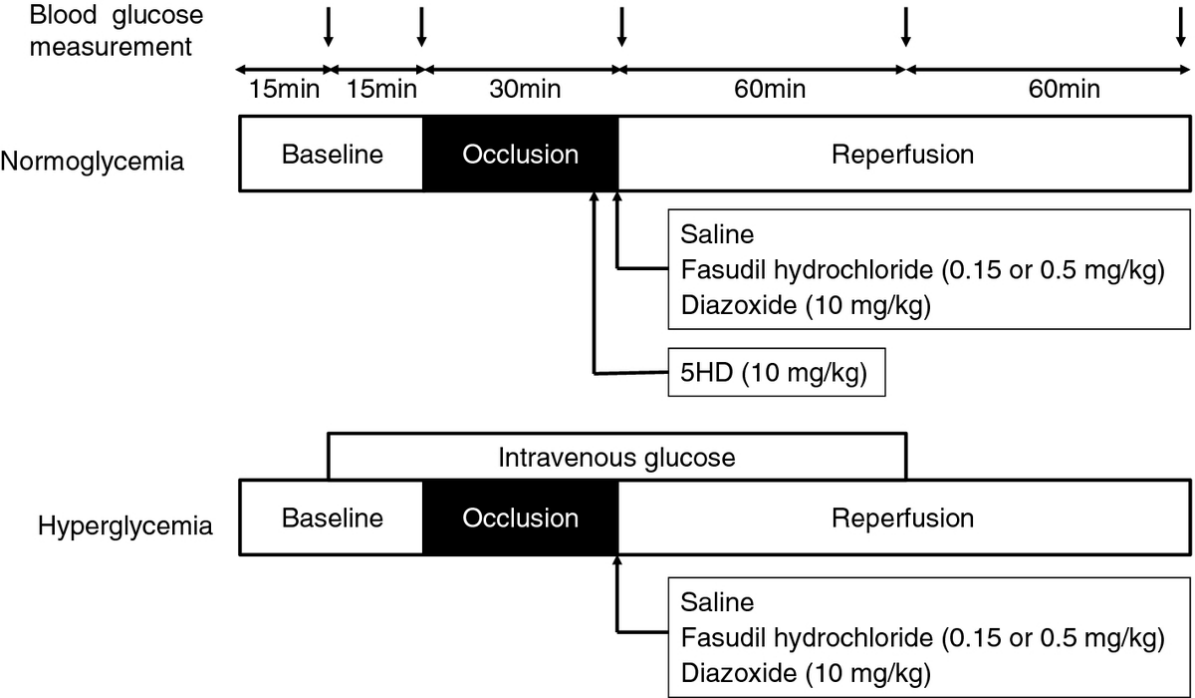
**A schematic illustration of the experimental protocols**.

The classification of experimental groups is shown in Table 1, and the experimental design is illustrated in Figure 1. All rats underwent 30-min LAD occlusion followed by 2-h reperfusion. The rats were randomly assigned to one of 10 groups (n = 10 for each). Six of them were under normoglycemic conditions, and 4 of them were under hyperglycemic conditions. The hyperglycemia was produced with continuous infusion of 50% glucose in water starting at 15 min before LAD occlusion and lasting until 60 min after reperfusion. The target hyperglycemia was 350 mg/dl.

**Table 1:** The classification of experimental groups

Groups	Treatments
CON	Saline, i.v. bolus just before reperfusion.
LF	Low-dose fasudil (0.15 mg/kg), i.v. bolus just before reperfusion.
HF	High-dose fasudil (0.5 mg/kg), i.v. bolus just before reperfusion.
DIA	Diazoxide (10 mg/kg), i.v. bolus just before reperfusion.
HG	Saline, i.v. bolus just before reperfusion, and continuous infusion of 50% glucose in water starting at 15 min before LAD occlusion and lasting until 60 min after reperfusion. The target blood glucose concentration was 350 mg/dl.
LF + HG	Same as group HG except for low-dose fasudil (0.15 mg/kg) in place of saline.
HF + HG	Same as group HG except for low-dose fasudil (0.5 mg/kg) in place of saline.
DIA + HG	Same as group HG except for diazoxide (10 mg/kg) in place of saline.
5HD	5HD (10 mg/kg), i.v. at 5 min before reperfusion followed by saline, i.v. bolus just before reperfusion.
HF + 5HD	Same as group 5HD except for high-dose fasudil (0.5 mg/kg) in place of saline.

In 4 normoglycemic groups, rats received saline as control (group CON), low-dose fasudil (0.15 mg/kg; group LF), high-dose fasudil (0.5 mg/kg; group HF) or diazoxide (10 mg/kg; group DIA) by i.v. bolus just before reperfusion.

In 2 normoglycemic groups, rats received 5HD, at 10 mg/kg, by i.v. bolus at 5 min before reperfusion followed by saline (group 5HD) or high-dose fasudil (group HF + 5HD) by i.v. bolus just before reperfusion.

In 4 hyperglycemic groups, rats received saline (group HG), low-dose fasudil (group LF + HG), high-dose fasudil (group HF + HG) or daizoxide (group DIA + HG).

The dose of fasudil was set on the basis of the clinical dosage for cerebral vasospasm after subarachnoid hemorrhage [20] as determined in our preliminary study. The dose of diazoxide was set on the basis of our preliminary study, which showed that 10 mg/kg but not 3 mg/kg diazoxide reduces infarct size (data not shown). The dose of 5HD was set on the basis of a previous study [19].

### Blood glucose measurement

Blood samples were collected to measure blood glucose in each group. The blood glucose concentration was determined by the glucose oxidase method using a blood glucose meter (Glutest Ace, Sanwa Kagaku Kenkyusho, Nagoya, Japan). Samples were taken before starting glucose infusion, just before ischemia, just after starting reperfusion, and at 1 and 2 h after starting reperfusion.

### Determination of infarct size

Myocardial infarct size was measured as previously described [21]. Briefly, at the end of each experiment, the LAD was reoccluded, and patent blue dye was administered intravenously to stain the normal region of the left ventricle (LV), and the heart was rapidly excised. LV tissue was isolated and cut into approximately ten cross-sectional pieces of equal thickness. The nonstained LV area at risk (AAR) was separated from surrounding blue-stained LV normal zone, and both regions were separately incubated at 37°C for 15 min in 1% TTC in 0.1 M phosphate buffer adjusted to pH 7.4. The tissues were fixed overnight in 10% formaldehyde. AAR and blue-stained LV normal zone region were weighed for determination of AAR/LV. TTC stains living tissue a deep red color, but necrotic tissue is TTC-negative and appears white within the AAR slices. Each slice was scanned at 1,200 dpi with a commercial scanner (Canoscan LiDE 60; Canon, Japan), and infarcted and noninfarcted areas were measured using an image analysis program. Myocardial infarct size is expressed as a percentage of the AAR.

### Statistical analysis

Statistical analyses of hemodynamic data and blood glucose concentrations within and between groups were performed with analysis of variance for repeated measures followed by Dunnett's test. Inter-group differences in body weight, age, LV weight, AAR weight, the ratio of AAR to LV and the ratio of infarct size to AAR were analyzed using one-way analysis of variance followed by the Student-Newman-Keuls test. Statistical significance was defined as *P *< 0.05. All values are expressed as mean ± SD. Statistical analysis was performed using SPSS 15.0 software (SPSS Japan, Tokyo, Japan) or GraphPad Prism 5.0 (GraphPad Software, San Diego, CA).

## Results

### Hemodynamics

There were no significant differences in body weight or age among the groups. Hemodynamic data for heart rate (HR) and mean blood pressure (MBP) are shown in Table 2. There were no significant differences either within groups or among groups in HR or MBP at any measurement point.

**Table 2:** Systemic hemodynamic

		Baseline	Occlusion 20 min	Reperfusion 20 min	1 h after reperfusion	2 h after reperfusion
CON	HR	112 ± 15	114 ± 20	120 ± 13	109 ± 15	101 ± 14
	MAP	423 ± 30	420 ± 34	425 ± 36	410 ± 38	407 ± 35
LF	HR	114 ± 12	119 ± 12	113 ± 14	104 ± 9	96 ± 9
	MAP	431 ± 28	427 ± 30	415 ± 34	405 ± 35	393 ± 35
HF	HR	107 ± 30	111 ± 28	106 ± 26	114 ± 25	115 ± 18
	MAP	408 ± 37	381 ± 57	395 ± 38	404 ± 36	392 ± 52
DIA	HR	104 ± 16	100 ± 20	97 ± 21	106 ± 28	106 ± 28
	MAP	403 ± 62	399 ± 66	388 ± 79	393 ± 69	400 ± 61
5HD	HR	104 ± 16	100 ± 20	102 ± 18	114 ± 25	110 ± 24
	MAP	394 ± 44	384 ± 52	385 ± 50	370 ± 32	407 ± 53
HF + 5HD	HR	108 ± 24	107 ± 27	112 ± 28	95 ± 27	99 ± 20
	MAP	404 ± 69	392 ± 51	392 ± 60	384 ± 69	397 ± 47
HG	HR	107 ± 20	91 ± 15	116 ± 25	113 ± 19	104 ± 15
	MAP	409 ± 33	403 ± 34	409 ± 36	396 ± 30	397 ± 40
LF + HG	HR	101 ± 12	93 ± 16	96 ± 19	106 ± 20	110 ± 17
	MAP	406 ± 42	388 ± 58	386 ± 60	398 ± 55	408 ± 53
HF + HG	HR	100 ± 29	104 ± 27	115 ± 25	108 ± 24	89 ± 30
	MAP	407 ± 65	407 ± 28	383 ± 64	376 ± 64	380 ± 76
DIA + HG	HR	106 ± 24	104 ± 16	102 ± 21	97 ± 18	104 ± 23
	MAP	389 ± 48	398 ± 53	396 ± 30	389 ± 45	383 ± 58

Data are mean ± SD, and n = 10 for each.

HR = heart rate (beats/min); MAP = mean arterial pressure (mmHg); CON = control; LF or HR = 0.15 or 0.5 mg/kg fasudil hydrochloride, DIA = 10 mg/kg diazoxide; 5HD = 5-hydroxydecanoic acid; HG = hyperglycemia.

### Infarct size

**Figure 2:**
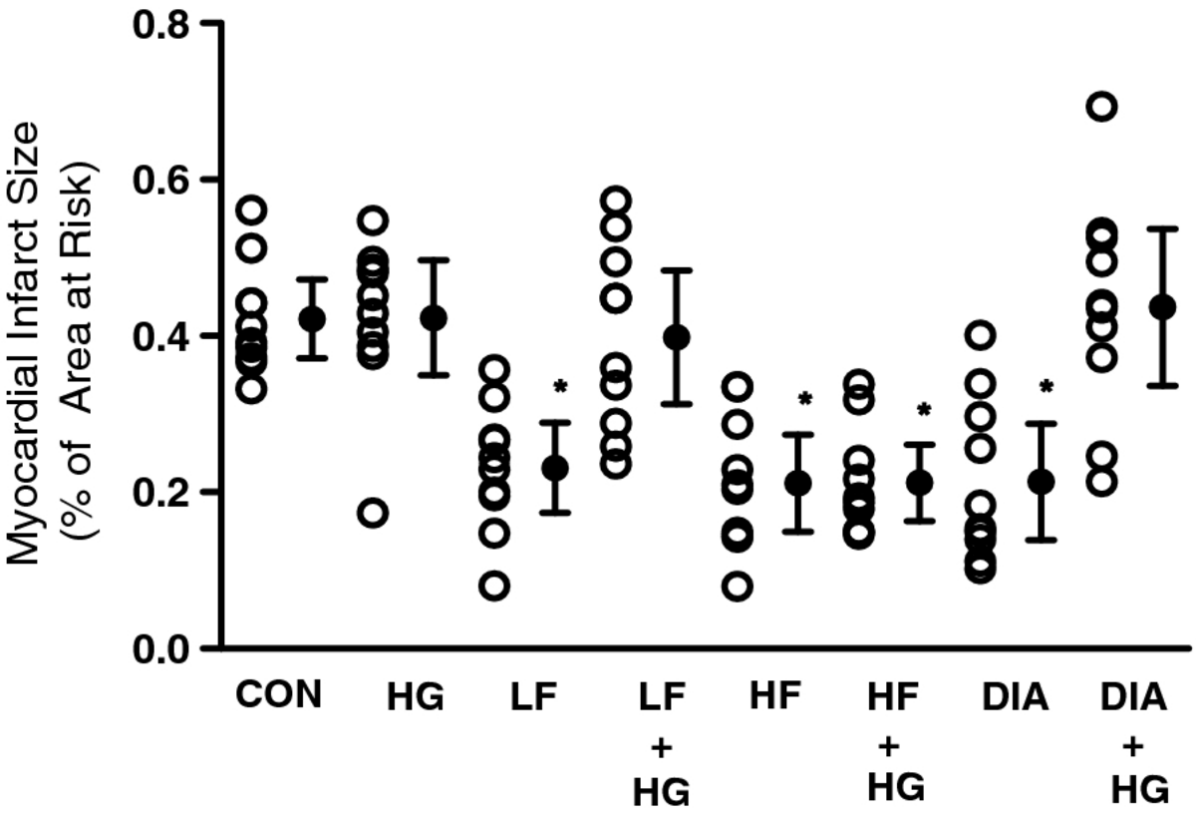
**Myocardial infarct size expressed as a percentage of the left ventricular area at risk**. Hyperglycemia (HG) group was administered 50% glucose during occlusion and until 1 h after reperfusion. CON received saline just before reperfusion; LF or HF, 0.15 or 0.5 mg/kg fasudil hydrochloride; and DIA, 10 mg/kg diazoxide. Data are mean ± SD and n = 10 for each. *Significantly (*p *< 0.05) different from control.

**Figure 3:**
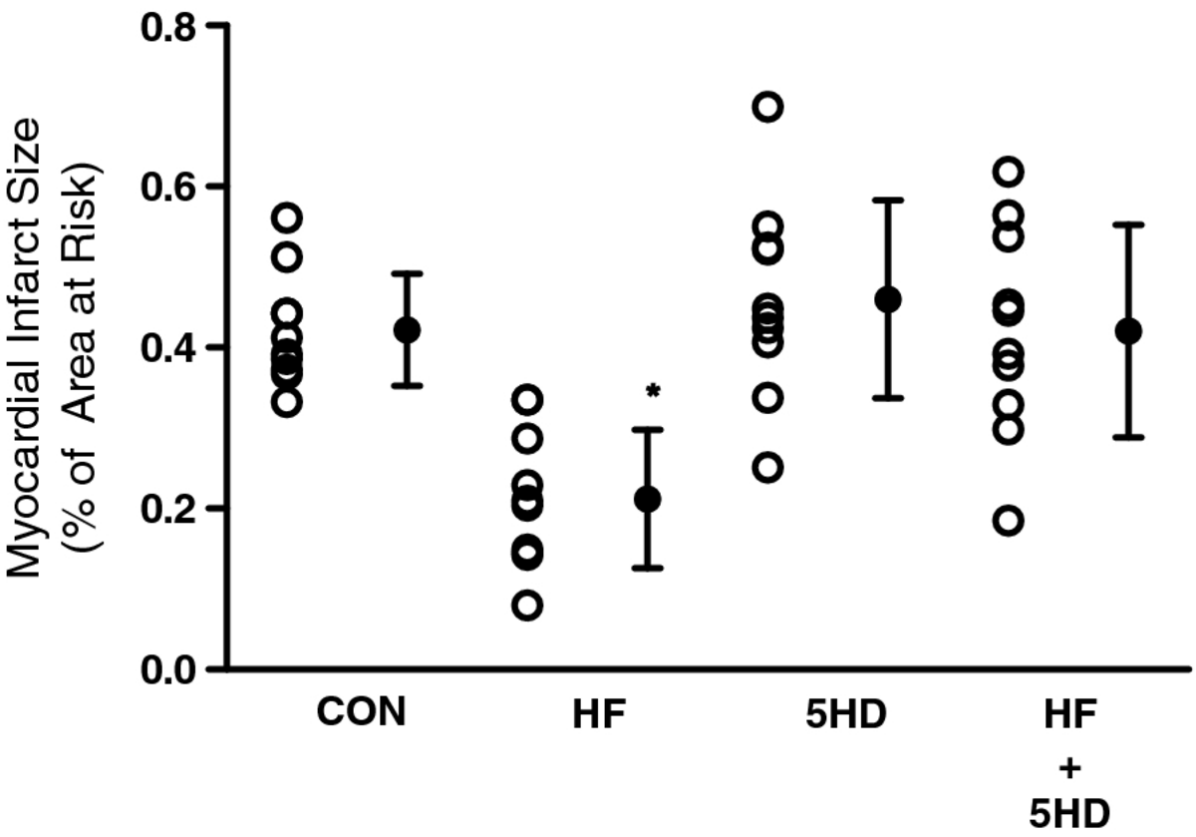
**Myocardial infarct size expressed as a percentage of the left ventricular area at risk**. 5HD group was administered 10 mg/kg 5-hydroxydecanoic acid at 5 min before reperfusion. CON received saline just before reperfusion; HF, 0.5 mg/kg fasudil hydrochloride. *Significantly (*p *< 0.05) different from control.

There were no significant differences in LV weight, AAR weight or the ratio of AAR to total LV mass among the groups (Table 3). Infarct sizes expressed as a percentage of AAR are shown in Figures 2, 3. In groups LF, HF and DIA, the infarct sizes were significantly reduced compared with that in group CON (Figure 2). In groups LF + HG and DIA + HG, the infarct sizes were similar to that in group CON (Figure 2). In contrast, the infarct size in group HF + HG was significantly less than that in group CON. In group HF + 5HD, the infarct size was similar to that in group CON (Figure 3).

**Table 3:** Left ventricular area at risk and infarct size

	AAR/LV (%)	IS/AAR (%)
CON	51 ± 9	42 ± 7
LF	46 ± 9	23 ± 8*
HF	52 ± 9	21 ± 9*
DIA	46 ± 7	21 ± 10*
5HD	48 ± 8	46 ± 12
HF + 5HG	44 ± 7	42 ± 13
HG	43 ± 13	42 ± 10
LF + HG	50 ± 9	40 ± 11
HF + HG	45 ± 7	21 ± 13*
DIA + HG	48 ± 9	44 ± 14

Date are mean ± SD, and n = 10 for earch.

CON = control; LF or HF = 0.15 or 0.5 mg/kg fasudil hydrochloride; DIA = 10 mg/kg diazoxide; 5HD = 5-hydroxydecanoic acid; HG = hyperglycemia.

*Significantly (*p *< 0.05) different from control.

### Blood glucose concentrations

As shown in Table 4, blood glucose concentrations at baseline did not differ among the groups. Blood glucose concentrations did not change significantly over time in normoglycemic groups. In hyperglycemic groups, blood glucose concentrations at just before ischemia, just after starting reperfusion and 1 h after starting reperfusion were significantly higher than those at baseline and 2 h after reperfusion. There were no significant differences in blood glucose concentrations among the hyperglycemic groups at any measured point.

**Table 4:** Blood glucose concentration (mg/dl)

	Baseline	Before ischemia	Just after reperfusion	1 h after reperfusion	2 h after reperfusion
CON	88 ± 15	91 ± 19	112 ± 27	105 ± 32	101 ± 20
LF	95 ± 11	109 ± 14	122 ± 26	118 ± 26	100 ± 23
HF	89 ± 17	87 ± 13	111 ± 33	93 ± 35	95 ± 26
DIA	90 ± 14	88 ± 14	107 + 31	120 ± 35	107 ± 28
5HD	89 ± 12	85 ± 16	101 ± 28	108 ± 18	111 ± 28
HF + 5HD	87 ± 8	86 ± 9	105 ± 24	101 ± 26	104 ± 24
HG	92 ± 12	349 ± 32*^†^	360 ± 50*^†^	353 ± 28*^†^	100 ± 39
LF + HG	92 ± 13	338 ± 24*^†^	378 ± 57*^†^	327 ± 29*^†^	105 ± 42
HF + HG	92 ± 12	341 ± 25*^†^	374 ± 52*^†^	337 ± 47*^†^	110 ± 28
DIA + HG	91 ± 13	349 ± 22*^†^	350 ± 53*^†^	353 ± 28*^†^	106 ± 37

Data are mean ± SD, and n = 10 for each.

CON = control; LF or HF = 0.15 or 0.5 mg/kg fasudil hydrochloride; DIA = 10 mg/kg diazoxide; 5HD = 5-hydroxydecanoic acid; HG = hyperglycemia.

*Significantly (*p *< 0.05) different from baseline.

†Significantly (*p *< 0.05) different from control.

## Discussion

The present results show that fasudil induces postconditioning against myocardial infarction via m-KATP channels in the rat, and that hyperglycemia prevents fasudil- and diazoxide-induced postconditioning, whereas high-dose fasudil can restore cardioprotection even under hyperglycemia.

In the present results, both high and low doses of fasudil produced equivalent reductions of myocardial infarct size under normoglycemia. However, hyperglycemia completely abolished the protective effect of the low dose, but not the high dose of fasudil. Fasudil is clinically used in doses of 30 mg/30 min intravenous infusion three times a day for cerebral vasospasm after subarachnoid hemorrhage [20], and 60 mg/60 min intravenous infusion twice daily for acute ischemic stroke [22]. Thus, the high dose used in the present study is relevant to the clinical dose, and it is indicated that fasudil at a clinical dose could induce PostC even under hyperglycemia.

Kersten et al. [12] showed that PreC evoked by 2.5 mg/kg but not 5 mg/kg diazoxide was blocked by hyperglycemia, and they assumed that the blockage of m-KATP channels was the underlying mechanism of the lack of myocardial protection by volatile anesthetics under acute hyperglycemia. Recently our group reported that hyperglycemia raised the threshold of levosimendan-induced PostC [23], and it is suggested that levosimendan-induced PostC involves the activation of m-KATP channels [24]. In the present results, low-dose fasudil- and diazoxide-induced PostC were attenuated by hyperglycemia, and 5-HD inhibited the fasudil-induced PostC. Therefore, it is likely that fasudil-induced PostC is mediated by m-KATP channels, which could be attenuated by hyperglycemia.

Zaho et al. [6] reported that the peri-conditioning stimulus of hydroxyfasudil was abrogated by glibenclamide, a non-selective KATP channel blocker, and suggested that activation of KATP channels could be mediated by Rho-kinase inhibition. Although some studies on PostC that targeted KATP channels generated negative results [25,26], a number of studies have revealed an obligatory role of m-KATP channels in both ischemic and pharmacological PostC [27-31]. Wolfrum et al. [4] reported that the Rho-kinase inhibition led to the activation of PI3K/Akt/eNOS pathway and PreC. Hamid et al. [5] showed that inhibition of Rho-kinase by Y27632, a structurally unrelated inhibitor of hydroxyfasudil, at reperfusion onset limited infarct size through an Akt/eNOS-dependent mechanism. Consequently, it is suggested that Rho-kinase acts as a negative regulator of PI3K activation, and modulates the protective effect via PI3K/Akt/eNOS pathway on myocardial PreC and PostC. Some kinds of pharmacological PostC were shown to depend on PI3K/Akt/eNOS pathway with a target downstream of m-KATP channels [27,28]. Therefore, it is likely that fasudil could induce the opening of m-KATP channels by up-regulating PI3K/Akt/eNOS pathway, resulting in induction of PostC.

Rho kinase activity is involved in diabetic endothelial dysfunction, and regulation of Rho kinase signaling is important for cellular function, such as contraction, mortality, proliferation, apoptosis [32]. Correspondingly, Rho-kinase is known to be activated in ischemic myocardium, and inhibition of Rho-kinase activity is a matter of great interest on myocardial protection against ischemic reperfusion injury [33]. However, there are few reports on the effect of hyperglycemia on the myocardial Rho-kinase activity during ischemia-reperfusion. It was shown that exposure of endothelial cells, renal glomerulus and cardiac fibroblasts to hyperglycemia increases Rho-kinase activity [34-37], and that the Rho-kinase activity occurred in a glycemic concentration-dependent manner [34,37]. Furthermore, the protective effect of Rho-kinase inhibitor depends on the dose under hyperglycemia [34,35,37]. Thus, it is likely that hyperglycemia could increase the myocardial Rho-kinase activity during ischemia-reperfusion, and raise the threshold of fasudil-induced PostC.

Hyperglycemia is an important predictor and a risk factor of cardiovascular morbidity and mortality [7-9], and pharmacological cardioprotection against ischemia-reperfusion injury is impaired by hyperglycemia, independent of diabetes [10-16]. PostC is considered to be more feasible than PreC for clinical application, and would be useful in unpredictable myocardial ischemia-reperfusion injury. Nevertheless, there are only a few reports concerning pharmacological PostC under hyperglycemia. Raphael et al. [11] showed that hyperglycemia inhibited PostC provided by isoflurane, and that the effect was mediated via modulation of PI3K/Akt/eNOS signaling. Huhn et al. [10] reported that sevoflurane-induced PostC was blocked by hyperglycemia, but the inhibition of mitochondrial permeability transition pore with cyclosporine A could reverse this loss of cardioprotection. Therefore, the present report is the first describe about Rho-kinase inhibitor-induced PostC under hyperglycemia.

Przyklen et al. [38] reported that diabetic hearts were refractory to ischemic PostC, but the effect in the type 1 diabetic model was reversed by restoration of normoglycemia. Whereas, Drenger et al. [27] showed that the protective effect of ishemic and sevoflurane PostC were not relived by insulin-induced normoglycemia in diabetic rats. Tsang et al. [39] reported that it was necessary to increase the ischemic PreC stimulus to achieve the threshold for cardioprotection and a critical level of Akt phosphorylation to mediate myocardial protection in diabetes. Furthermore, rats with type 1 diabetes for 4 weeks showed ischemic PreC weaker than normal rats, but there was no cardioprotection in the rats for 8 weeks [40]. These finding suggested that the threshold for cardioprotection in diabetes will gradually increase with the prolongation of hyperglycemic time. Therefore, it is likely that the threshold of PostC in the diabetic animals or in animals exposed to longer periods of hyperglycemia is higher than acute hyperglycemia.

Marinovic et al. [41] reported that sarcolemma KATP (s-KATP) channels act as an effector and m-KATP channels play a dual role as a trigger and an effector in cardioprotection by isoflurane-induced PreC against oxidative stress. Furthermore, Yang et al. [42] showed that KR31761, a new KATP channel opener, exerted PreC in rats, and both 5-HD and HMR-1098, an s-KATP channel blocker, prevents the cardioprotective effects. Chen et al. [43] reported that s-KATP channels were decreased in diabetes and under hyperglycemia, and it lead to result in desensitization of some KATP channel drugs. Thus, there is possibility that cardiprotection via s-KATP channels is attenuated under hyperglycemia. However, mechanism for s-KATP channels dependent PreC and PostC is not clarified, and there are few reports about s-KATP channels involved with rho-kinase. Further research is needed to elucidate the molecular mechanisms contributing to this cardioprotective effect.

## Conclusion

Fasudil induces PostC against myocardial infarction in the rat. This protective effect of fasudil is mediated by activation of m-KATP channels. Although hyperglycemia raises the threshold of fasudil-induced PostC, high-dose fasudil could preserve the protective effect.

## Competing interests

The authors declare that they have no competing interests.

## Authors' contributions

TI performed experiments, contributed to discussion, and drafted the manuscript. SC participated in the design and coordination of the study, contributed to discussion and reviewed/edited the manuscript. UH and SM helped carry out in vivo experiments. TM participated in the design and coordination of the study. KS supervised research to discussion and reviewed/edited the manuscript. All authors read and approved the final manuscript.
